# Supplementary material for: The interactome of intact mitochondria by cross-linking mass spectrometry provides evidence for coexisting respiratory supercomplexes
Source: Mol Cell Proteomics. 2017 Dec 8;17(2):216–32. doi: 10.1074/mcp.RA117.000470 (PMC5795388; doi:10.1074/mcp.RA117.000470)
Supplement: Supplemental Data [file supp_RA117.000470_133922_0_supp_23978_fzffwf.zip › spectra_annotation/mito_spectra_annotation/134-1-3-1-1-1.pdf]

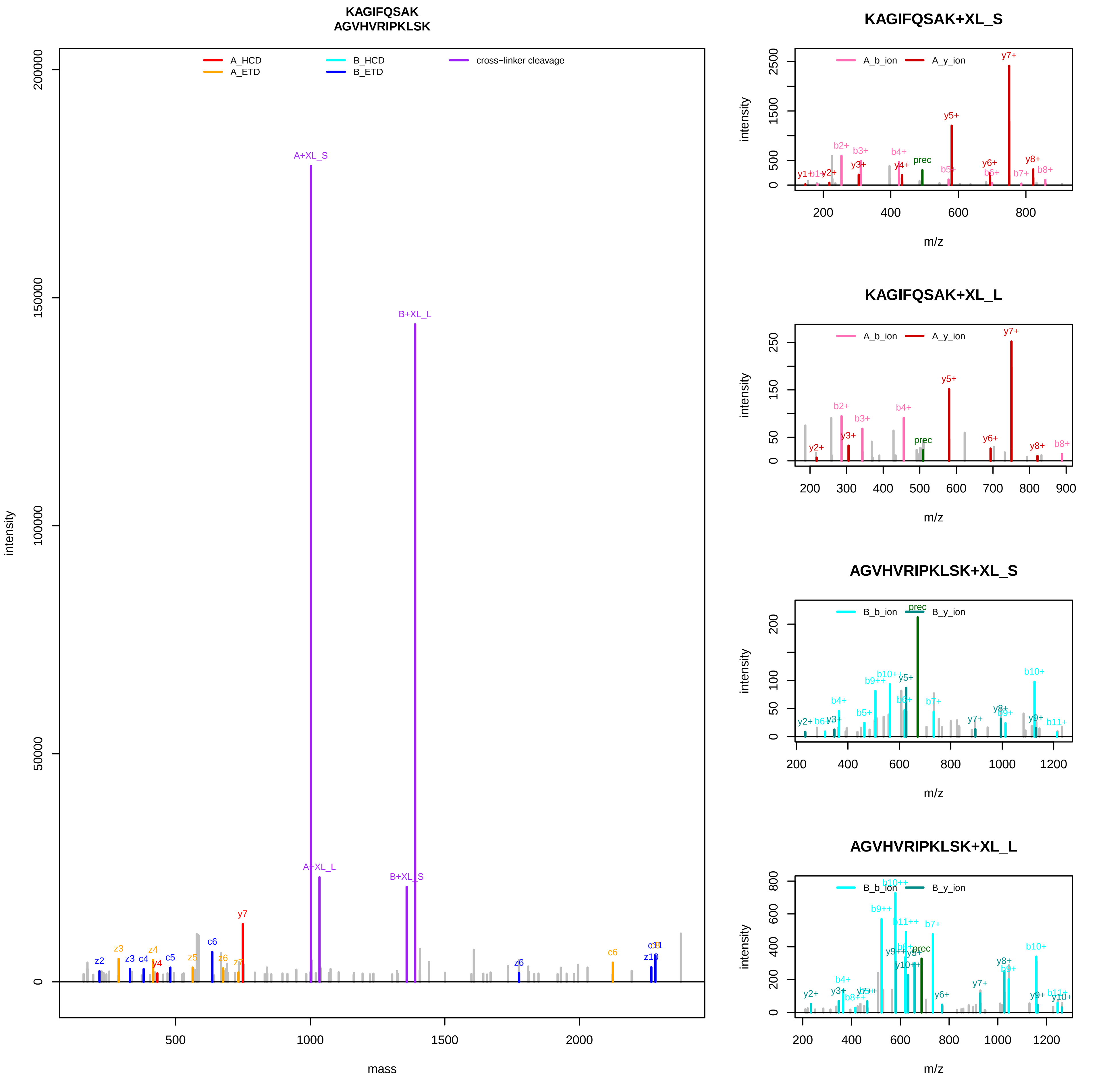
KAGIFQSAK
AGVHVRIPKLSK
A_HCD
A_ETD
B_HCD
B_ETD
cross−linker cleavage
A+XL_S
B+XL_L
A+XL_L
B+XL_S
y7
c6
z6
c6
z10
intensity
mass
0
50000
100000
150000
200000
500
1000
1500
2000
KAGIFQSAK+XL_S
A_b_ion
A_y_ion
y7+
y5+
prec
y8+
y6+
m/z
KAGIFQSAK+XL_L
A_b_ion
A_y_ion
y7+
y5+
b4+
b2+
b3+
prec
y6+
y8+
b8+
m/z
AGVHVRIPKLSK+XL_S
B_b_ion
B_y_ion
prec
b10+
b10++
b9++
y5+
b4+
b7+
y7+
y8+
y9+
b11+
m/z
AGVHVRIPKLSK+XL_L
B_b_ion
B_y_ion
b10++
b9++
b11++
b7+
prec
b10+
y8+
y7+
y6+
b4+
m/z
